# Supplementary material for: Altered microRNA Transcriptome in Cultured Human Liver Cells upon Infection with Ebola Virus
Source: Int J Mol Sci. 2021 Apr 6;22(7):3792. doi: 10.3390/ijms22073792 (PMC8038836; doi:10.3390/ijms22073792)
Supplement: Supplementary file 1 [file ijms-22-03792-s001.zip › Supplementary_File/C_ GO_Analysis_Results/16-30nt_go_Reston-24h-Huh7_vs_Control-24h-Huh7_down.mature_mirna_targets/BP_EnrichmentScoreDotPlot.pdf]

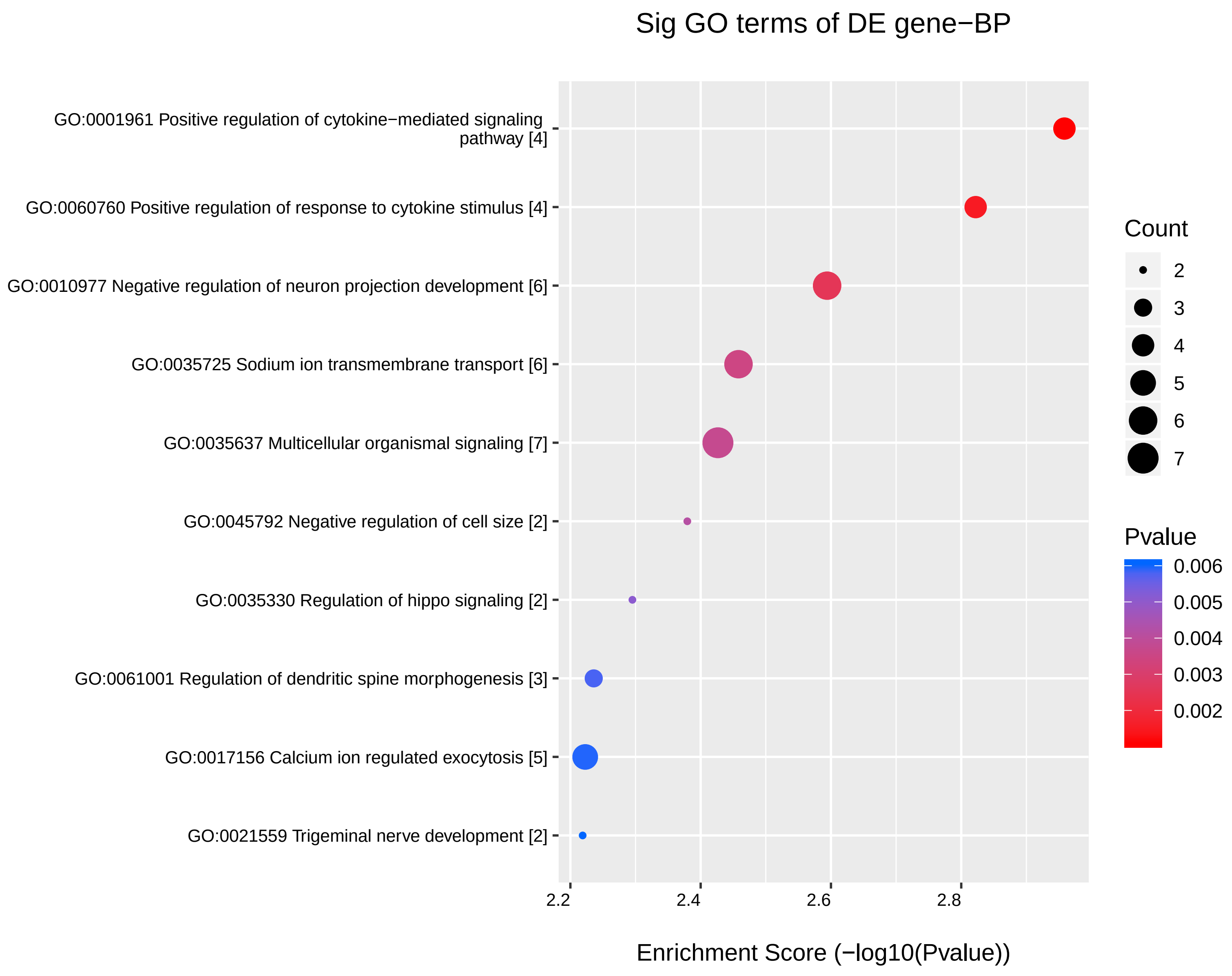
Sig GO terms of DE gene−BP
GO:0001961 Positive regulation of cytokine−mediated signaling pathway [4]
GO:0060760 Positive regulation of response to cytokine stimulus [4]
GO:0010977 Negative regulation of neuron projection development [6]
GO:0035725 Sodium ion transmembrane transport [6]
GO:0035637 Multicellular organismal signaling [7]
GO:0045792 Negative regulation of cell size [2]
GO:0035330 Regulation of hippo signaling [2]
GO:0061001 Regulation of dendritic spine morphogenesis [3]
GO:0017156 Calcium ion regulated exocytosis [5]
GO:0021559 Trigeminal nerve development [2]
2.2
2.4
2.6
2.8
Enrichment Score (−log10(Pvalue))
Count
2
3
4
5
6
7
Pvalue
0.006
0.005
0.004
0.003
0.002
